# Measurement of body composition in response to a short period of overfeeding

**DOI:** 10.1186/1880-6805-33-29

**Published:** 2014-09-10

**Authors:** Hiroyuki Sagayama, Yu Jikumaru, Akiko Hirata, Yosuke Yamada, Eiichi Yoshimura, Mamiko Ichikawa, Yoichi Hatamoto, Naoyuki Ebine, Akira Kiyonaga, Hiroaki Tanaka, Yasuki Higaki

**Affiliations:** 1 Graduate School of Sports and Health Science, Fukuoka University, 8-19-1 Nanakuma, Jounanku, Fukuoka 814-0180, Japan; 2 Research Fellow of Japan Society for the Promotion of Science, 5-3-1 Koujimachi, Chiyoda-ku, Tokyo 102-0083, Japan; 3 Fukuoka University Institute for Physical Activity, 8-19-1 Nanakuma, Jounan-ku, Fukuoka 814-0180, Japan; 4 Section of Energy Metabolism, Department of Nutritional Science, National Institute of Health and Nutrition, 1-23-1 Toyama, Shinjuku-ku, Tokyo 162-8636, Japan; 5 Faculty of Environmental and Symbiotic Sciences, Prefectural University of Kumamoto, 3-1-100, Tsukide, Higashi-ku, Kumamoto 862-8502, Japan; 6 Faculty of Health and Sports Science, Doshisha University, 1-3 Tatara Miyakodani, Kyotanabe, Kyoto 610-0394, Japan; 7 Faculty of Sports and Health Science, Fukuoka University, 8-19-1 Nanakuma, Jonan-ku, Fukuoka 814-0180, Japan

**Keywords:** overfeeding, body composition, total body water

## Abstract

**Background:**

Obesity and overweight are increasing in prevalence in developed countries as a result of changing dietary habits and a lack of physical activity. The purpose of the present study was to evaluate the changes in body composition during short-term overfeeding using the three-component model, which is composed of fat mass (FM), total body water (TBW), and fat-free dry solids (FFDS).

**Methods:**

Ten healthy men completed 3 days of overfeeding during which they consumed 1,500 kcal/day more energy than consumed in their normal diets. Body composition was evaluated at three time points: the day before and after their normal diets and the day after the 3-day overfeeding diet.

**Results:**

Before and after their normal diets, there were no significant differences in body weight and composition, but after 3 days of overfeeding, body weight, TBW, and FFDS increased 0.7, 0.7, and 0.2 kg, respectively (*P* <0.0001). There was no significant difference in FM between the normal and overfeeding diets.

**Conclusion:**

This study suggests that TBW gain contributes to weight gain following a short-term overfeeding.

## Background

Obesity and overweight are increasing in prevalence in developed countries as a result of changing dietary habits and a lack of physical activity (PA) [1-4]. Both conditions are caused by a chronic imbalance between energy intake (EI) and expenditure (EE). A positive balance between EI and EE is a key factor in weight gain caused by overfeeding or decreasing activity energy expenditure (AEE). Most of the accumulation of excess energy is stored as lipid, mainly triglycerides, with overfeeding [5]. Lipid is ideal for long-term energy store, with little water accumulation in humans. Therefore, huge quantities of triglycerides can be stored with increasing adipocyte size and number during positive energy balance [6,7].

Several previous studies have suggested that EI exceeding EE for 2 to 8 weeks led to increased fat mass (FM) [8-10]. Moreover, the concept of non-exercise activity thermogenesis (NEAT) seems important in energy balance regulation as in the study, which overfed 16 non-obese subjects with 4.2 MJ/day for 56 days; changes in NEAT directly predicted resistance to FM gain from overfeeding [8]. Additionally, there is an association between weight gain and sedentary time during 3 days of overfeeding [11]. Thus, AEE is the most important component of energy expenditure to maintain body weight and composition during overfeeding. However, there is little detailed evidence of changes in body composition when AEE is maintained during overfeeding. Additionally, there is poor information regarding body composition during short-term overfeeding. Therefore, we hypothesized that fat mass would not be gained during overfeeding if AEE could be maintained. Thus, the purpose of the present study was to evaluate changes in body composition during short-term overfeeding using the three-component model, which includes FM, total body water (TBW), and fat-free dry solids (FFDS).

## Methods

Ten healthy, non-obese Japanese men participated in this study (mean ± standard deviations; age = 23.1 ± 1.6 years; height = 171.7 ± 3.6 cm; body weight = 63.6 ± 4.5 kg; and body mass index = 21.6 ± 1.3 kg/m^2^). All subjects lacked chronic diseases that could affect body composition, metabolism, or daily PA. The subjects were invited to attend an informational meeting and those interested in participating in the study provided written informed consent. The study protocol was approved by the Ethics Committee of Fukuoka University (10-12-02).

**Figure 1:**
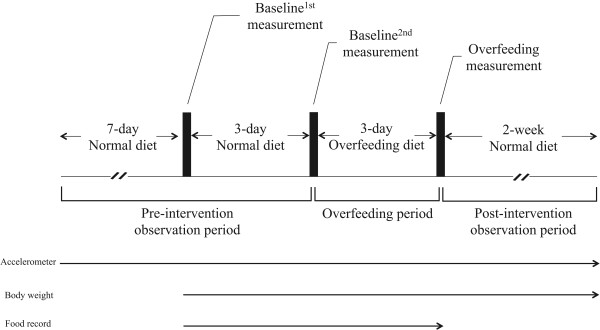
Study protocol.

The experimental design of the study is shown in Figure 1. Body composition was evaluated at three time points: the day before the 3-day normal diet of the survey period (Baseline^1st^ [BL^1st^]); the day after the 3-day normal diet of the survey period (this day is the same measurement before overfeeding) (Baseline^2nd^ (BL^2nd^)); and the day after the overfeeding diet period (Overfeeding (OF)). Subjects measured their own body weights twice daily for the 6 days (in the morning fasting and again before going to bed) from BL^1st^ to OF. Additionally, subjects measured their own body weights (in the morning fasting) for 2 days during the postintervention observation period and for 2 weeks following completion of OF.

The normal EI survey was defined over a 3-day period (between BL^1st^ and BL^2nd^ measurement). The overfeeding EI survey defined a 3-day period following a normal diet (between BL^2nd^ and OF measurement). We informed all subjects about their normal EI to maintain that level of EI. During the 3-day overfeeding period, subjects were overfed with a diet supplying 1500 kcal per day more energy than the 3-day normal EI. Diets were self-selected during normal and overfeeding periods. Excess EI during the overfeeding period was selected based on the energy information shown on food packages.

### Body composition measurement

Subjects came to the laboratory early in the morning on the 3 measurement days after a 12-h fast. All body composition measurements were carried out at the same time each morning after urination and defecation. Height was measured to the nearest 0.1 cm with a stadiometer. Body weight was measured using a calibrated balance beam scale (Shinko Denshi Vibra Co., Ltd., Tokyo, Japan) to the nearest 0.01 kg, with the subjects wearing only light undergarments. Hydrostatic weighing and stable isotope dilution method estimated body density and total body water. Subjects were administered these stable isotopes using the following protocol: ^2^H_2_O, H_2_^18^O, and ^2^H_2_O for BL^1st^, BL^2nd^, and OF measurements, respectively. Our previous study provides details regarding the evaluation of body composition using the three-component model [12].

### Physical activity and energy intake

Daily AEE was evaluated using a triaxial accelerometer (Panasonic Electric Works Co., Ltd., Osaka, Japan) [13], which was attached to the waist for about 1 month until the end of the study (from 1 week before BL^1st^ until the postintervention observation period finished). Subjects were instructed to refrain from vigorous exercise and to maintain their lifestyle for about 1 month. The data of baseline PA were obtained for 7 days with the exception of the first 3 days since attaching the triaxial accelerometer. Subjects strictly maintained baseline PA by checking levels of PA using the triaxial accelerometer during the overfeeding period (between BL^2nd^ and OF measurement). If the non-wear activity time of the accelerometer exceeded 3 h in a day, with the exception of the time for taking a bath and sleeping, that day was excluded from the analysis.

All foods and beverages were weighed using a portable digital scale (KS-232; Dretec Co. Ltd., Saitama, Japan) during the BL^2nd^ and OF measurement periods (3 days). Furthermore, a survey of food intake was conducted using both self-reporting methods and visual records obtained using a digital camera or a mobile phone with a camera. A well-trained registered dietitian checked calculated nutrients from the diet records with the photographs. EI was measured daily from a week before the BL^1st^ until the OF measurement. All diet records were analyzed using a computerized nutrient analysis program (Excel Eiyoukun Ver. 4.5; Kenpakusha, Tokyo, Japan).

### Statistical analysis

The results are presented as means ± standard deviations. Comparisons between two groups (BL^1st^ versus BL^2nd^ and BL^2nd^ versus OF) were made with the paired *t*-test using Microsoft Excel 2010 from Microsoft Office 2010 (Microsoft Corp., Redmond, WA, USA). The intraclass correlation coefficient (ICC) and the coefficient of variation (CV) were used to test the reproducibility of body weight, % fat, FM, FFDS and TBW measured by the three-component models. Values of ICC above 0.7 were considered as having excellent reproducibility. An alpha of 0.05 was used to denote statistical significance.

## Results

### Body compositions

We first evaluated body composition and measurement reproducibility. All components of body composition did not change between BL^1st^ and BL^2nd^ (Table 1; body weight = -0.2 ± 0.5 kg, *P* = 0.17;% fat = -0.1 ± 0.5%, *P* = 0.49; FM = -0.1 ± 0.4 kg, *P* = 0.36; TBW = -0.1 ± 0.4 kg, *P* = 0.56; FFDS = 0.0 ± 0.4 kg, *P* = 0.71). The ICC for all body composition values was above 0.9. The CV for all body composition values was less than 3%.

**Table 1:** Change in body composition, coefficient of variation and intraclass correlation coefficient during normal diet

	**Baseline**^**1st**^	**Baseline**^**2nd**^	**CV (%)**	**ICC**
Body weight (kg)	63.6 ± 4.5	63.4 ± 4.1	0.5	0.996
Percent of fat (%)	14.5 ± 3.0	14.4 ± 3.0	2.2	0.991
Fat mass (kg)	9.3 ± 2.5	9.2 ± 2.5	2.2	0.994
Fat-free dry solid (kg)	15.5 ± 0.8	15.5 ± 0.7	1.3	0.946
Total body water (kg)	38.7 ± 2.1	38.7 ± 2.0	0.6	0.992

There were no significant differences between BL^1st^ and BL^2nd^ for each of these.CV, coefficient of variation; ICC, intraclass correlation coefficient.

**Figure 2:**
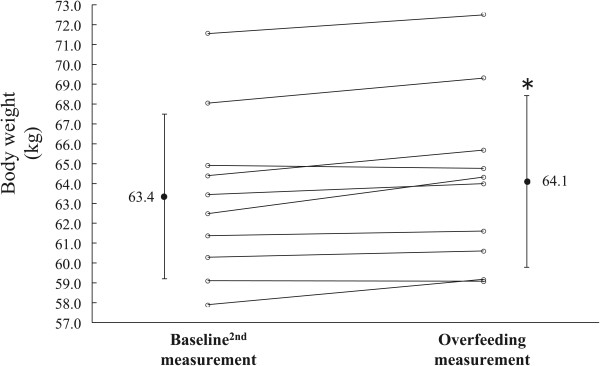
**Changes in body weight.** *P <0.01 versus Baseline^2nd^ measurement.

**Figure 3:**
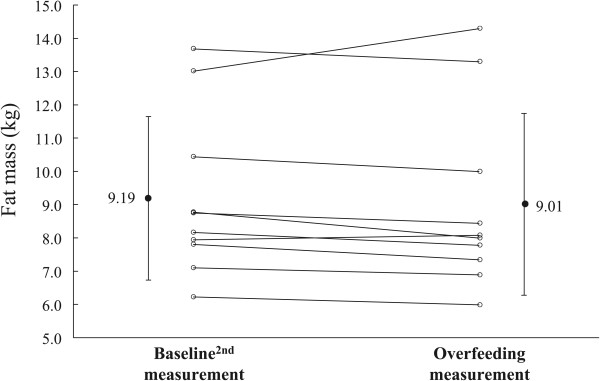
Changes in fat mass.

**Figure 4:**
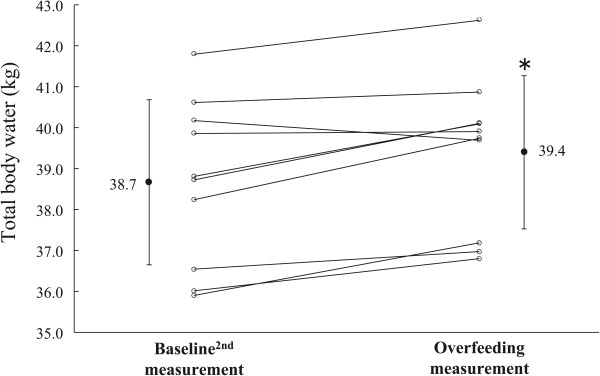
**Changes in total body water.** *P <0.01 versus Baseline^2nd^ measurement.

Body weight, TBW, and FFDS increased during OF compared with BL^2nd^ measurements (Table 2 and Figures 2, 3, and 4; body weight = 0.7 ± 0.5 kg; TBW = 0.7 ± 0.4 kg; FFDS = 0.0 ± 0.4 kg, *P* <0.0001). There were no significant differences in FM and % fat between the BL^2nd^ and OF measurements (Table 2). Subjects measured their body weights during the postintervention period. All subjects returned to BL^2nd^ body weights within 2 weeks (5.0 ± 4.9 days).

**Table 2:** Changes in body composition during overfeeding

	**Baseline**^**2nd**^	**Overfeeding**
Body weight (kg)	63.4 ± 4.1	64.1 ± 4.3^**^
Percent of fat (%)	14.4 ± 3.0	13.9 ± 3.3
Fat mass (kg)	9.2 ± 2.5	9.0 ± 2.7
Fat-free dry solid (kg)	15.5 ± 0.7	15.7 ± 0.7^*^
Total body water (kg)	38.7 ± 2.0	39.4 ± 1.9^**^

**P <0.01, *P <0.05 versus Baseline^2nd^ measurement.

### Physical activity and energy intake

During the normal and overfeeding periods, there were no significant differences in levels of PA (1.6 ± 0.2 and 1.6 ± 0.1, respectively) and AEE (835 ± 261 and 875 ± 240 kcal/day, respectively) (Table 3). Energy, weights of diets, and sodium intakes increased during the overfeeding period (*P* <0.05). Fat intake significantly increased and carbohydrate intake decreased during overfeeding, affecting the protein, fat, and carbohydrate rate (PFC rate). There was no significant increase in the protein rate.

**Table 3:** Changes in physical activity and energy intake before and after overfeeding

	**Baseline**^**2nd**^	**Overfeeding**
Energy intake (kcal/day)	2452 ± 297	3906 ± 427^**^
Protein intake (kcal/day)	296 ± 44	450 ± 73^**^
Fat intake (kcal/day)	695 ± 96	1379 ± 258^**^
Carbohydrate intake (kcal/day)	1421 ± 262	2004 ± 288^**^
Protein rate (%)	12.2 ± 1.7	11.5 ± 1.1
Fat rate (%)	28.1 ± 4.4	35.3 ± 5.1^**^
Carbohydrate rate (%)	58.0 ± 4.9	51.4 ± 5.2^**^
Weight of diet (g/day)	2557 ± 615	3353 ± 724^**^
Sodium intake (mg/day)	4131 ± 1013	6079 ± 1856^**^
Physical activity level	1.6 ± 0.2	1.6 ± 0.1
Active energy expenditure (kcal/day)	835 ± 261	875 ± 240

**P <0.01 versus Baseline^2nd^ measurement.

## Discussion

The major finding of this study is that TBW is the main component of body composition affected during overfeeding when AEE is maintained at the level during normal diets. Our results suggested that the increased body weight for 3 days of overfeeding was mostly TBW. There were no significant differences in body weight or composition at BL^1st^ and BL^2nd^. The ICC values ranged from 0.946 to 0.996 in the body composition measurements in the current study, which is in agreement with previous studies [14]. Thus, the results and methods are thought to be of excellent reproducibility.

The overfeeding of 1,500 kcal per day over 3 consecutive days led to increased body weight, TBW, and FFDS, though there were no significant increases in FM and % fat. Participants were asked to overeat an average of 4,500 kcal for 3 days, and were able to do so successfully. Assuming that an FM of 1 kg is equivalent to 7,000 kcal and that 85% of the EI would be accumulated as fat in this case, FM was expected to increase by 0.5 kg. However, FM did not increase. In a previous study of overfeeding an excess of 1,000 kcal per day for 8 weeks, increases in body weight, and FM were reported (weight, 1.4 to 7.2 kg; FM, 0.36 to 4.23 kg) [8]. Assuming that an FM of 1 kg is equivalent to 7,000 kcal and that 85% of the EI would be accumulated as fat in this case, the FM was expected to increase by 6.8 kg. Unexpectedly, body weight and FM in the previous study were not increased as much as expected. Moreover, there were large individual differences in the increases in FM and body weights, as pointed out by some researchers. In particular, the study suggested individual NEAT and sedentary time were different during overfeeding [8,11]. We, therefore, instructed subjects in the present study to maintain PA during overfeeding. As a result, the AEE during the 3-day overfeeding period is similar to the AEE during the normal diet period. Thus, PA is not the only factor involved in the lower-than-expected increase in FM during overfeeding. Other factors could include an increase in diet-induced thermogenesis [15] and increased lipid catabolism [16]. The unexpected large interindividual variation in the efficiency of weight gain with overfeeding shows that adaptive thermogenesis and other factors are still an issue. Further, the accelerometers worn at the waist may not be able to evaluate arm and leg movement as a component of activity.

Body weight (on average 0.7 kg) increased as well as TBW (on average 0.7 kg) during the 3 days of overfeeding. Increased TBW could be the result of ingestion of an excess amount of sodium during overfeeding. After the ingestion of dietary sodium, there is a subsequent rise in plasma sodium, and to maintain fluid homeostasis thirst is stimulated, which promotes fluid consumption [17]. In a previous study that compared a high and low salt diet over 50 days, the high-salt diet group had a greater increase in weight compared with the low-salt group [18]. Moreover, dietary sodium is positively associated with fluid consumption and predicted sugar-sweetened beverage consumption [19]. Following the increase in EI, sodium intake and TBW increased in our study. Thus, water and sugar-sweetened beverage intake could be associated with these increases. The temporary accumulation of sodium may result in increased body weight as a result of transient overfeeding. Glycogen storage, which is known to increase body weight during carbohydrate overfeeding [20], may be another factor to consider. The molecular fraction of glycogen is hydrated by water molecules in a ratio of approximately 1:3, and structurally contains an abundant amount of water [21-24]. Therefore, it has the possibility to contribute to the increase seen in TBW.

The content of the diet was self-selected during the normal and overfeeding periods of our study. The EI of macronutrients during that period significantly increased in terms of PFC. However, the PFC rate was only significantly increased in terms of fat intake. These results suggested that it is possible to consume more energy from fat during self-selected overfeeding. In a previous study comparing overfeeding of high carbohydrate and high fat diets of equal energy, body weight and FM significantly increased in both diet groups. However, that study did not detect a between-group difference [9]. If self-selected overfeeding for 3 days involves a high carbohydrate diet, this may result in the promotion of more weight gain because of increased storage of glycogen and water.

The increased body weight returned to the baseline body weight over an average of 5 days, though there were individual differences (0 to 14 days). When subjects were free to follow their regular lifestyles during the postintervention period, their body weights reduced relatively early. These results support the hypothesis that the component of increased body weight in our study was a result of increased TBW.

A limitation of our study is that a diet survey and information regarding bowel movements were not measured during the postintervention period. The EI during the postintervention observation period is a matter of speculation; differences in each subjects’ EIs were considered a possible effect of the rapid weight loss. Additionally, the presence, absence, and amount of bowel movements are a reflection of weight cycling during a short period of overfeeding. Additionally, when the fat mass increase started is unknown; thus, further studies are needed to clarify these factors.

## Conclusions

TBW is the main component in overfeeding when AEE is maintained at levels seen during normal feeding.

## Abbreviations

AEE: activity energy expenditure; BL: baseline; CV: coefficient of variation; EE: energy expenditure; EI: energy intake; FFDS: fat-free dry solid; FM: fat mass; ICC: intraclass correlation coefficient; NEAT: non-exercise activity thermogenesis; OF: overfeeding; PA: physical activity; PFC rate: protein, fat and carbohydrate rate; TBW: total body water; % fat: percent of fat.

## Competing interests

The authors state that there are no personal conflicts of interest in the present study.

## Authors’ contributions

HS, EY, YY, YH, AK, and YH, conception and design of the study; HS, YJ, EY, YY, MI, and YH, acquisition of data; HS, YJ, AH, EY, YY, MI, HT, and YH, analysis and interpretation of data; HS, YJ, YY, and NE drafting the manuscript; HS, YJ, EY, YH, HT, NE and YH, revising the manuscript; and all of the authors approved the final version of the manuscript.
